# Wearable Wide-Range Strain Sensors Based on Ionic Liquids and Monitoring of Human Activities

**DOI:** 10.3390/s17112621

**Published:** 2017-11-14

**Authors:** Shao-Hui Zhang, Feng-Xia Wang, Jia-Jia Li, Hong-Dan Peng, Jing-Hui Yan, Ge-Bo Pan

**Affiliations:** 1 College of Chemistry and Environmental Engineering, Changchun University of Science and Technology, Changchun 130022, China; shzhang2016@sinano.ac.cn; 2 Suzhou Institute of Nano-Tech and Nano-Bionics, Chinese Academy of Sciences, Suzhou 215123, China; jjli2015@sinano.ac.cn (J.-J.L.); hdpeng2012@sinano.ac.cn (H.-D.P.)

**Keywords:** wearable, wide-range, strain sensor, ionic liquids

## Abstract

Wearable sensors for detection of human activities have encouraged the development of highly elastic sensors. In particular, to capture subtle and large-scale body motion, stretchable and wide-range strain sensors are highly desired, but still a challenge. Herein, a highly stretchable and transparent stain sensor based on ionic liquids and elastic polymer has been developed. The as-obtained sensor exhibits impressive stretchability with wide-range strain (from 0.1% to 400%), good bending properties and high sensitivity, whose gauge factor can reach 7.9. Importantly, the sensors show excellent biological compatibility and succeed in monitoring the diverse human activities ranging from the complex large-scale multidimensional motions to subtle signals, including wrist, finger and elbow joint bending, finger touch, breath, speech, swallow behavior and pulse wave.

## 1. Introduction

Wearable sensors have attracted great attention due to their fascinating efficacy, and achieved considerable progress along with the development of flexible and stretchable electronics [1,2]. Such sensors require the capability of detecting physiological signals as small as pulses and as large as human motions. Thus, high sensitivity and broad sensing range are indispensable to these wearable sensors [3]. However, conventional strain gauge sensors can only detect a narrow range of strain with low gauge factors (GF) due to the rigid nature of constituent metal and semiconductor materials [4]. Various promising methods have been purposed to achieve a highly stretchable sensor [5,6,7,8]. In general, the strategies of embedding conductive filler into an elastomeric matrix, and fabricating a micro/nano-pattern on the surface of a device were purposed to enhance stretchability [8,9,10,11,12,13,14]. Despite the high performance of these devices, local material delamination or cracking and a narrow sensing range still restrict widespread application [15]. 

Recently, ionic conductor and liquid metal with lower Young’s modulus than elastomeric supports, which theoretically eliminated the crack or delamination, were used as filler materials to construct wearable sensors [16,17,18,19]. These sensors showed high stretchability and good sensitivity. However, the high cost and surface tension for liquid metal [20,21] and poor electro-mechanical stability of aqueous electrolytes [16,22] limited their further application in wearable electronics. Moreover, ionic liquids (ILs) were used to fabricate wearable sensors combining the 3D-printed mold, screen printing method and soft molds, exhibiting high stretchability and wide-range sensing [23,24,25,26,27,28]. However, a high-cost complex fabrication process [23] and the trade-off relationship between the high sensitivity and broad sensing range [26,27] still limited the application in detecting physiological signals as small as pulses and as large as human motions. Moreover, the vast majority of the reported sensors were single-function, incapable of sensing multiple forms of mechanical deformation. In addition, ILs had tunable physicochemical properties owing to different anion-cation pairs with millions of possibilities [29], Therefore, the different types of ILs-based sensors could provide different characteristics. 

Herein, the highly stretchable and sensitive wearable sensors based on ILs were fabricated using a trimmed PDMS mold and Eco-flex polymers. The higher conductivity of the 1-ethyl-3-methylimidazolium bis(trifluoromethylsulfonylmide) ([EMIM][TFSI]) ILs was used [23,26]. The higher current provided a stable system and a smaller jamming signal. The as-obtained sensor exhibited excellent stretchable and bending properties, a wide sensing range (from 0.1% to 400%) and high sensitivity with GF of 7.9, which reduced the trade-off between the high sensitivity an broad sensing range, showing wider application in monitoring diverse human activities, especially in subtle signal detection. Moreover, the flexible sensors were sensitive to temperature and pressure with the limit of detection of 28 Pa. Importantly, the sensors could accurately identify behaviors, from accurate speech recognition, pulse wave, breath and swallow behavior to vigorous human motions such as finger and wrist movement capturing. The easy fabrication process and multifunctional characteristics gave the stretchable sensor potential application in wearable electronics.

## 2. Results and Discussion

**Figure 1:**
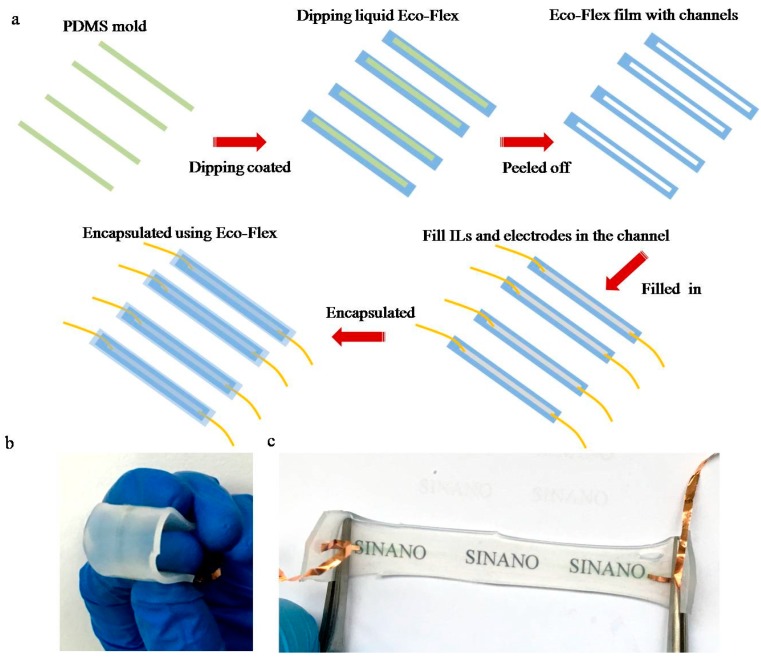
(**a**) Schematic of the process fabrication of sensor. (**b**) Photograph of the bending sensor. (**c**) Photograph of the stretching sensor.

The fabrication process of the sensor based on ILs is schematically illustrated in Figure 1a with detailed description presented in the experimental section. With ILs as conductive liquids contained in an Eco-flex silicon elastomeric cover, a shape-adaptive ionic conductor unit was fabricated to detect the pressure/strain/bending.

The as-fabricated sensor showed excellent flexibility, high transparency and stability (Figure 1b,c, Figures S1 and S2). This sensor was also waterproof, which was sensitive to water temperature, indicating potential application in temperature detection (Figure S3). Increasing temperature could enhance the migration rate of ionic mobility, resulting in higher electrical conductivity. Moreover, the electrical characteristics of the sensor depended on the ILs species and the channel size. As shown in Figure S4, the current was increased by shortening the alkyl chain of imidazoliun cation or increasing the size of anion, which was similar to the reported literature [30]. Therefore, the [EMIM][TFSI] ILs with the higher conductivity were used. In addition, the current of the sensor decreased with the channel length and increased with the channel width (Figure S5). The resistance showed an opposite trend, which showed similar change trends as the conductor resistance equation:

(1) $$R=\rho \cdot \frac{L}{A}$$

where R is the initial resistance, ρ is the electrical resistivity, L is the length of the channel, and A is the cross sectional area of the channel.

**Figure 2:**
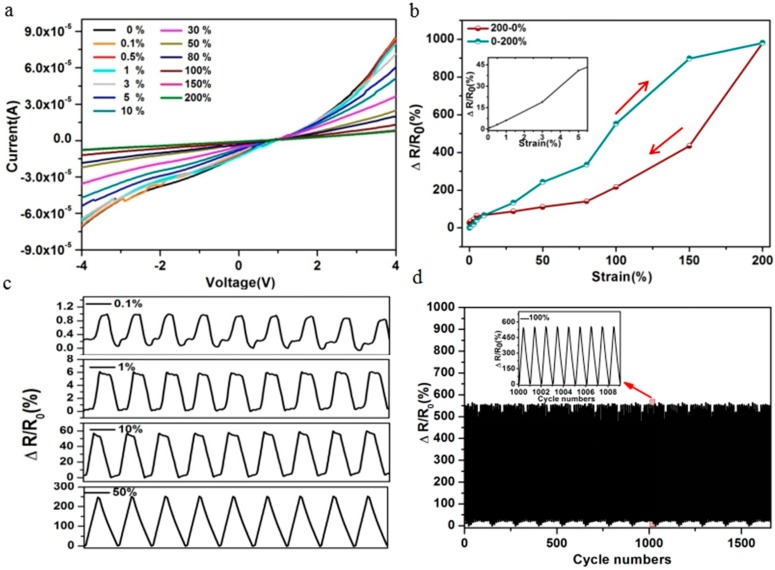
(**a**) The I-V curves under different strain. (**b**) The relative resistance change under different strain. (**c**) Plots of relative resistance change under the strain 0.1%, 1%, 10% and 50% at input voltage of 4 V. (**d**) The relative resistance change under stain 100% at input voltage of 4 V.

Figure 2 shows the stretchable properties of the sensor based on ([EMIM][TFSI]), where the length, width and height of the channel is 32 mm, 1.5 mm and 2 mm, respectively. It was seen that the current decreased with the increase of the strain (Figure 2a), which was attributed to the change of resistance in the channel. When the rubber band was deformed, the resistance of the sensor would change to a value described in the following equation:

(2) $$R_{X}=\rho \cdot \frac{L\left(\varepsilon +1\right)}{A_{X}}$$

where *R_X_* is the resistance under deformation, ε is the strain, and A_X_ is the cross-sectional area of the stretched channel (assuming the channels are uniformly deformed). Therefore, deformation such as stretching or bending result in a change of resistance. Herein, to clearly show the effects of the strain on the electrical characteristics, the relative resistance variation (ΔR/R_0_ = ((R − R_0_)/R_0_)) or relative current variation ((ΔI/I_0_ = ((I − I_0_)/I_0_)) were traced under various strain-loading conditions by maintaining constant voltage across the sensor device, where R_0_ and I_0_ are electrical properties without strain, and R and I are electrical properties under strain. The resistance increased monotonically with the strain increase from 0.1% to 200%. The largest sensing strain was 400%, which compared favorably to or far better than some reported results (Figure 2b and Figure S1) [3,7]. Noted that the ΔR/R_0_ linearly increased with strain increase (Figure S6**)** and the reverse process showed different variation trend, indicating that the hysteresis existed.

Gauge factor (GF), defined as (ΔR/R_0_)/ε, where ε was the strain, was the representative parameter to assess sensitivity. GF was calculated from the curve of ΔR/R_0_-ε (Figure 2b). The largest GF can reach 7.9 within 5% strain and 1.7 was obtained within the 100%, which was higher than some reported results [31,32]. The response current signals would be large for high conductive stain sensors, thus very small applied strains would be able to produce detectable signals. In the case of a low conductive strain sensor, the applied small strain may result in the small response current, which may not be identifiable. Therefore, the high conductivity of [EMIM][TFSI] and good contact between the ILs conductive layer and electrode may be main factors resulting in high sensitivity. Additionally, the sensor showed high sensitivity to the pressure, and the detection limit was 28 Pa (Figure S7). It was expected that pressure would lead to deformation of the channel (reduction in size), and thus an increase in the volume concentration of ions, which led to a decrease in the resistance of the device. The above results demonstrated that the strain sensor possessed good sensitivity for sensing electrical signal changes induced by strain or pressure.

The strain sensor showed fast switching and stable properties to wide-range strain between the stretching and releasing cycles. As shown in Figure 2c, the relative resistance was almost unchanged after several cycles, indicating higher stability and wide-range strain sensing. The response curves of the sensor measured after 1500 cycles under stain 100% at input voltage of 4 V is shown in Figure 2d. The output signals of the relative resistance change exhibited negligible change, indicating remarkable stability. Benefiting from high sensitivity at small strain range, the detection limit could be as small as 0.1%, and the output signal was highly reproducible at small strains (Figure 2c), accompanied by superb signal stability. Moreover, the sensor also showed a fast response. The stretching and releasing time was defined as the time required for the relative resistance change to increase from 10% to 90% of the peak value in a single on/off cycle, while the releasing time, vice versa, was about 0.18 s, and 0.65 s respectively (Figure S8), which was fast or comparable to reported results. The above results indicated that the sensor showed high stability, fast response and a desirable combination of high sensitivity at small strain and broad sensing range, which expanded its application in daily life, especially as a wide-range sensing human motion sensor.

**Figure 3:**
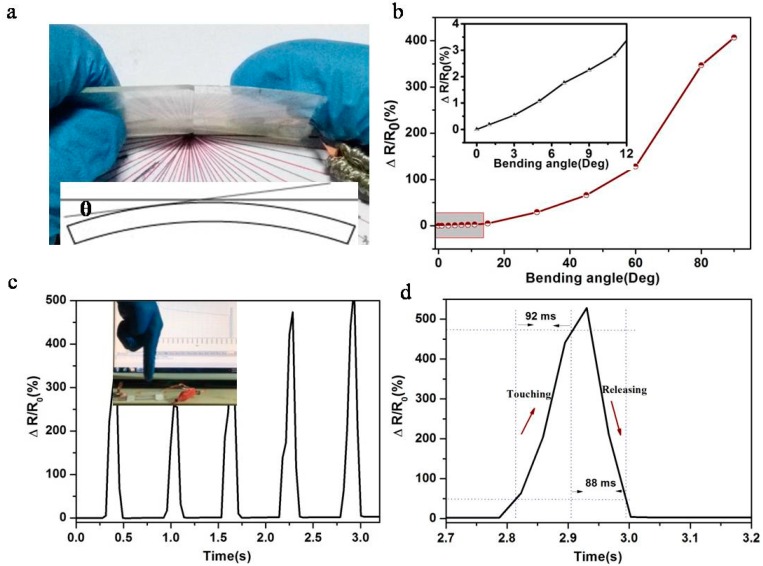
(**a**) Photograph of the bending sensor, Inset: the definition of bending angle θ in a schematic structure. (**b**) The relative resistance change under different bending angles at input voltage of 4 V. (**c**) The relative resistance with the finger touching. Inset: the photograph of finger touching at input voltage of 4 V. (**d**) The relative resistance change in one touching cycle at input voltage of 4 V. The touching and releasing time was defined to 90% high value.

To detect bending, we integrated the stain sensor on the PET substrate. The sensor inherited the superb flexibility of the PET upholder and the bending angle θ was defined in the inset of Figure 3a. We harnessed the bending-induced strain on the upper surface of the PET slab as a stimulus to the sensor, which in turns reflected the bending extent. Figure 3b shows the relative resistance change at different bending angles, which revealed a wide sensing range of bending angle up to 90°. Even with a small bending of 1°, an obvious relative resistance change was obtained, which corresponded to 0.05% bending strain [33], showing a prime bending strain sensitivity (Figure S9, Table S1) [31,34,35,36,37]. The outstanding properties in bending sensing enabled the sensor to monitor joint bending of humans and robots.

Except for tensile strain and bending, the strain sensor showed high sensitivity and fast response to touching. When a finger lightly touched the senor, the significant change of relative resistance was generated. Moreover, the response signal had a sharp peak instead of a state curve, which illustrated a fast response and no hysteresis with fast switching (Figure 3c). Here, the touching time was defined as the time required for the relative resistance change to increase from 10% to 90% of the peak value in a single on/off cycle, while the releasing time was vice versa. The touching and releasing time was 92 ms and 88 ms (Figure 3d), respectively, which was far better than reported results (Table S1) [32]. The excellent features in touching sensing would enrich the application of sensor in robot touching sensor. 

**Figure 4:**
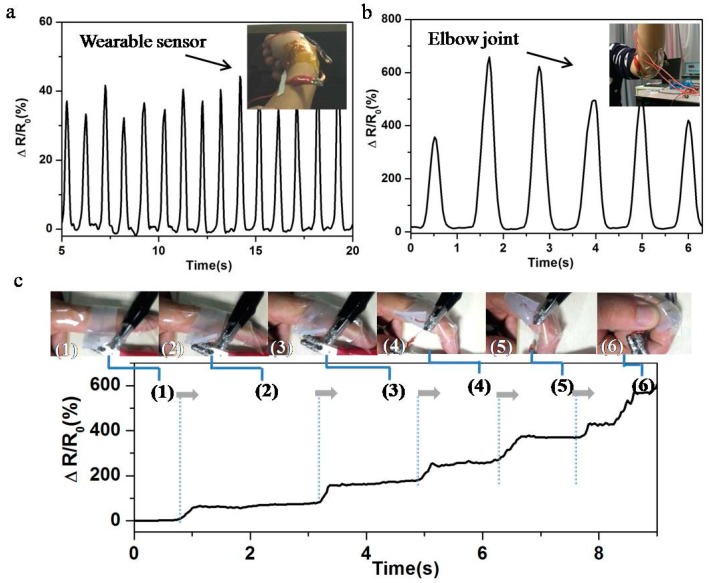
(**a**) The relative resistance change for monitoring of the wrist bending movement at input voltage of 4 V. Inset: the photograph of wearable sensor on the wrist joint. (**b**) The relative resistance change for monitoring of the elbow joint movement at input voltage of 4 V. Inset: the photograph of sensor on the elbow joint. (**c**) The relative resistance change of sensor monitoring the finger bending with different directions at input voltage of 4 V.

The great potential applications of wearable sensors toward the monitoring of diverse human activities were also investigated. Due to stretchable and biomedical properties, these sensors can be attached at different positions of human body. The strips of tape were attached on the edge of the sensor to restrict skin/body motions. Figure 4 and Figure S10 show the wearable sensor for the monitoring of large-scale human motions such as finger, wrist and elbow joint. As shown in insets of Figure 4a,b, wearable sensors were directly attached to the back of a person’s wrist or elbow joint. These human activities can be easily recorded with obvious and fast relative resistance changes. In addition, different directions of finger bending can be detected and distinguished through electrical signals generated by strain sensors (Figure 4c), which has potential application in detection of human or robot movements. 

**Figure 5:**
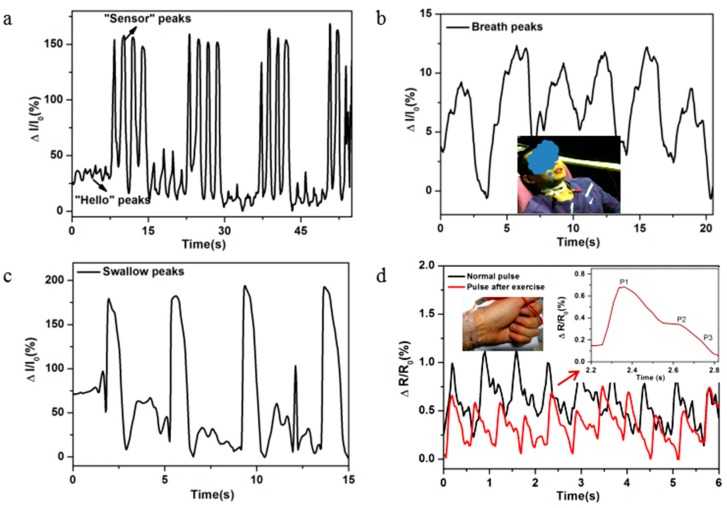
(**a**) The relative resistance changes of the wearable sensor at input voltage of 4 V when the wearer said “Hello” and “sensor”. (**b**) The relative resistance changes of the wearable sensor at input voltage of 4 V when wearer took a breath. Inset: the photograph of wearable sensor on the throat. (**c**) The relative resistance changes of the strain sensor at input voltage of 4 V when wearer swallowed slobber. (**d**) The relative resistance change as a function of time at input voltage of 4 V when monitoring the wrist pulse. Inset: magnified view of one particular waveform.

Besides large-scale motion monitoring, this strain sensor can be used to detect subtle physiological signals. First, the strain sensor was attached on the throat as shown in inset of Figure 5. The sensor showed remarkable signal changes with good repeatability when a speaker said “hello” and “sensor”, and the sensor could read the differences of different words (Figure 5a). Moreover, the sensors could detect breath/swallowing behaviors. When a human took a breath or swallowed saliva, the electrical signals of the wearable sensor exhibited obvious peaks (Figure 5b,c). These behaviors mainly resulted from complicated epidermis/muscles movements around throat during phonation or breath or swallowing. In particular, these unique properties exhibit potential in applications such as aiding speech rehabilitation training and human-machine interaction and the behavior training. Wrist pulse was a key physiological signal for determining arterial blood pressure and heart rate. As shown in Figure 5d, the wearable sensor could identify pulses, where waveforms could be detected with high fidelity. Two distinguished peaks and a late augmentation systolic shoulder could be clearly seen, which were due to blood pressure from the left ventricle contractions and the reflective wave from the lower body, thus showing potential application in disease detection. Moreover, this sensor clearly read the differences of amplitude and frequency before and after exercise. The normal pulse frequency was 80 beats per min with regular and repeatable pulse shape. After exercise, the pulse frequency increased to 110 beats per min, while the amplitude of pulse waveforms decreased obviously. High sensitivity of pulse detection exhibits potential application in the detection of health and disease.

## 3. Experimental Section

Materials: Polydimethylsiloxane (PDMS-184) was purchased from Dow Corning Corporation Midiand-Michigan USA. Eco-flex 0030 rubber was purchased from Shanghai Zhixin Tech Co., Ltd. (Shanghai, China). 1-ethyl-3-methylimidazoliumbis(trifluoromethyl-sulfonylmide) ([EMIM][TFSI]), 1-butyl-3-methylimidazoliumbis(trifluoromethylsul-fonylmide) ([BMIM][TFSI]), 1-octyl-3-methylimidazoliumbis(trifluoromethylsulfony-lmide) ([OMIM][TFSI]), 1-ethyl-3-methylimidazoliumtrifluoroacetate ([EMIM][TFAc]), 1-ethyl-3-methylimidazoliumdicyanamide ([EMIM][DCA]), 1-ethyl-3-methylimidazoliumacetate ([EMIM][Ac]) and 1-ethyl-3-methylimidazoliumtetrafluoroborate ([EMIM][BF_4_]) were purchased from Shanghai Chengjie Chem Co., Ltd. (Shanghai, China). These ILs with 99% purity were used directly without further purification.

Fabrication process: The PDMS pre-polymer mixed solution was first spin-coated on the clean silicon wafer at 350 rpm and heated at 80 °C for 2 h. The solidified PMDS film was cut into different sizes as a mold in the following process. The mixture of Eco-flex was then poured on the PDMS mold surface and solidified. The Eco-flex film was peeled off and the PDMS mold was removed, forming a small channel. Then, the ILs were filled in the small channel with copper foils attached as electrodes at both ends. To avoid forming a bubble, a non-invasive interface was formed between the ILs and the Eco-flex film. Finally, another cut layer of Eco-flex film covered the channel from one side carefully and the mixture solution of Eco-flex was poured on the surface of the covered film to seal the ILs and fix the electrodes. After solidification of the Eco-flex, the stretchable sensor was obtained.

Characterization: The characteristics of the strain sensor were measured by a displacement table (Z812B, Thorlabs, Newton, NJ, USA) and a controller (TDC001, Servo Motor Driver T-Cube, Newton, NJ, USA). The electrical properties were measured using the semiconductor meter Keithley 4200.

## 4. Conclusions

A flexible and stretchable strain sensor based on ILs was fabricated using the PDMS mold and simple sealing method. It was found that the electrical signals of as-obtained sensor depended on the ILs species and the channel size. A typical sensor possessed the combination of high sensitivity, with GF of 7.9, and broad workable range in strain sensing and bending sensing, which could detect fine and wide the strains as low as 0.1% and as high as 400%. Moreover, the wearable sensors exhibited the capability of multiple deformation forms including strain, bending and pressure, which exhibited their application in the detection of finger touching, finger, wrist and elbow joint movement, throat muscle movement and pulse waveforms. Another highlight was the easy, low-cost, scalable fabrication strategy, setting the stage for the practicable and widespread utilization as a wearable sensor. The versatile fabrication strategy, unique structure and excellent properties made them potential candidate in a new wearable sensor.

## Supplementary Materials

The following are available online at www.mdpi.com/1424-8220/17/11/2621/s1, Figure S1: (a) The I–V curves of the sensor before and after stretching 400%. (b) The photograph of the sensor and its resistance of sensor without stretching. (c) The photograph of the sensor and its resistance of sensor after 400% stretching. The sensor channel size (length 32 mm, width 1.5 mm, height 2 mm); Figure S2: The current change of the sensor with the increase of storage time; Figure S3: (a) The relative resistance changes of sensor before and after dipping into the water. (b) The relative current changes of sensor under different water temperature. The sensor channel size (length 32 mm, width 1.5 mm, height 2 mm); Figure S4: The I–V curves of devices based on different ILs. (a) Different anions, (b) cations with different alkyl chain length. The sensor channel size (length 32 mm, width 1.5 mm, height 2 mm); Figure S5: (a) The I–V curves of the sensors with different channel lengths (The channel width 1.5 mm, height 2 mm). (b) The I–V curves of the sensors with different channel widths (The channel length 32 mm, height 2 mm); Figure S6: The relationship of the relative resistance change to the stretch (λ = L/L_0_); Figure S7: The relative current changes of the sensor under different pressures. The sensor channel size (length 32 mm, width 1.5 mm, height 2 mm); Figure S8: The relative resistance change in one stretching cycle under stain 1% at input voltage of 4 V; Figure S9: The relative resistance change under different bending strain at input voltage of 4 V; Figure S10: The relative resistance change for monitoring of the finger joint movement at input voltage of 4 V. Inset: the photograph of sensor on the finger joint; Table S1: The comparing results of reported literature and this work.

## Abbreviations

PDMS-184	Polydimethylsiloxane
PET	Polyethylene terephthalate
[EMIM][TFSI]	1-ethyl-3-methylimidazoliumbis(trifluoromethyl-sulfonylmide)
[BMIM][TFSI]	1-butyl-3-methylimidazoliumbis(trifluoromethylsul-fonylmide)
[OMIM][TFSI]	1-octyl-3-methylimidazoliumbis(trifluoromethylsulfony- lmide)
[EMIM][TFAc]	1-ethyl-3-methylimidazolium trifluoroacetate
[EMIM][DCA]	1-ethyl-3-methylimidazolium dicyanamide
[EMIM][Ac]	1-ethyl-3- methylimidazolium acetate
[EMIM][BF_4_]	1-ethyl-3-methylimidazolium tetrafluoroborate
