# Supplementary material for: Wearable Wide-Range Strain Sensors Based on Ionic Liquids and Monitoring of Human Activities
Source: Sensors (Basel). 2017 Nov 14;17(11):2621. doi: 10.3390/s17112621 (PMC5712858; doi:10.3390/s17112621)
Supplement: Supplementary file 1 [file sensors-17-02621-s001.pdf]

Wearable wide-range strain sensor based on ionic liquids and monitoring of human activities

Shao-Hui Zhang[1,2], Feng-Xia Wang[2,*], Jia-Jia Li[2], Hong-Dan Peng[2], Jing-Hui Yan[1,*] and Ge-Bo Pan[2,*]

[1] College of Chemistry and Environmental Engineering, Changchun University of Science and Technology, 130022 Changchun, China; yjh@cust.edu.cn

[2] Suzhou Institute of Nano-tech and Nano-bionics, Chinese Academy of Sciences, Suzhou 215123, P. R. China; gbpan2008@sinano.ac.cn; fxwang2009@sinano.ac.cn

* Correspondence: gbpan2008@sinano.ac.cn; Tel.: +86-0512-62872663

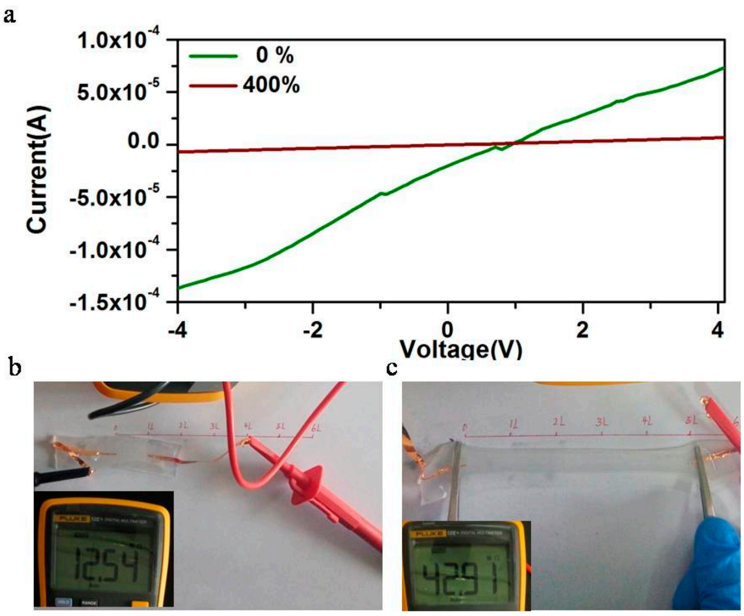

Figure S1. (a) The I-V curves of the sensor before and after stretching 400%. (b) The photograph of the sensor and its resistance of sensor without stretching. (c) The photograph of the sensor and its resistance of sensor after 400% stretching. The sensor channel size (length 32 mm, width 1.5 mm, height 2 mm).

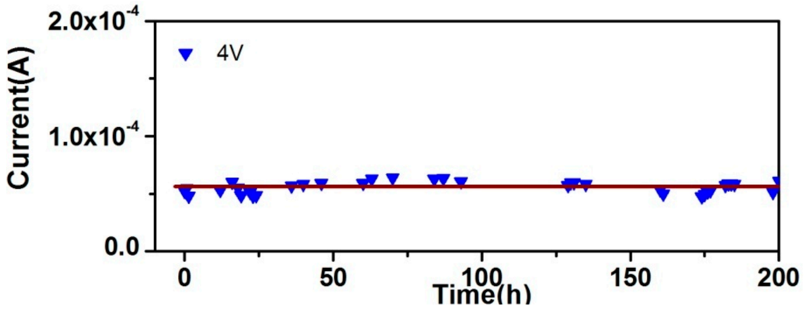

Figure S2. The current change of the sensor with the increase of storage time.

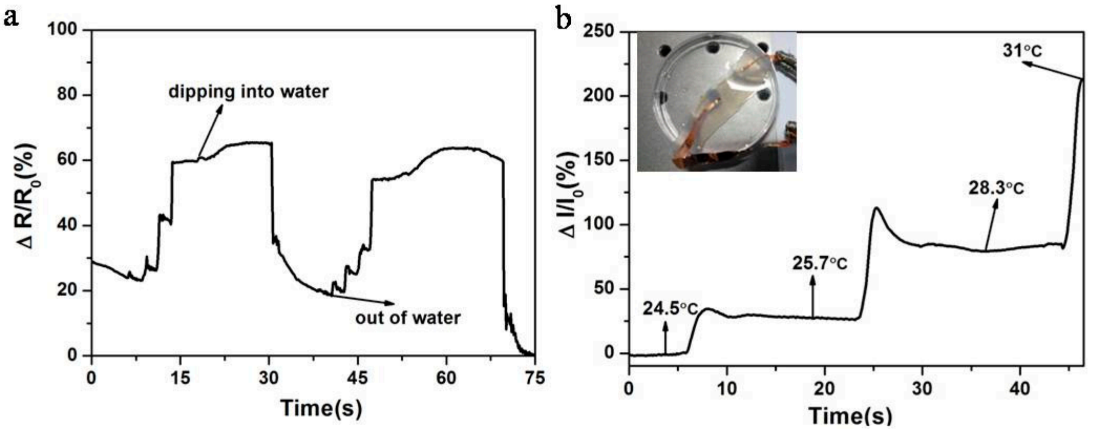

Figure S3. (a) The relative resistance changes of sensor before and after dipping into the water. (b) The relative current changes of sensor under different water temperature. The sensor channel size (length 32 mm, width 1.5 mm, height 2 mm).

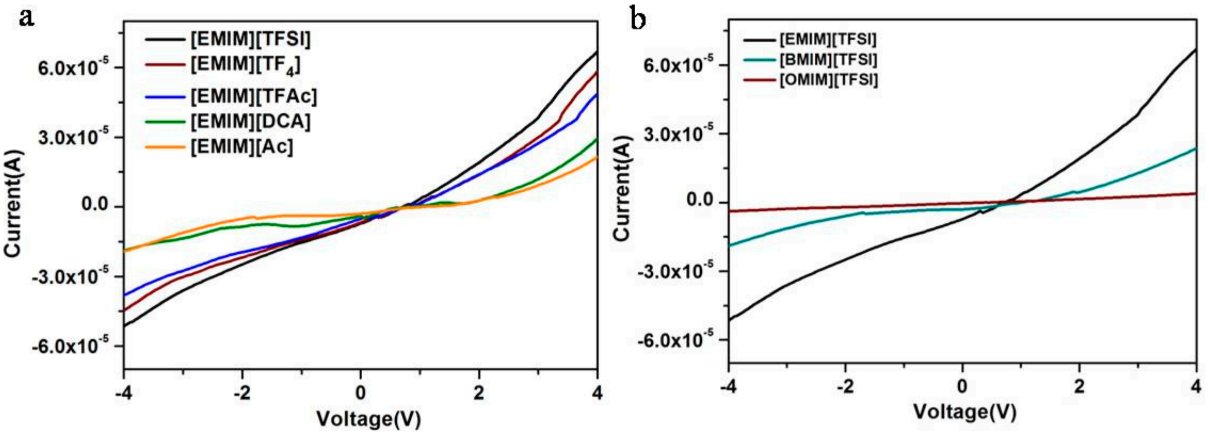

Figure S4. The I-V curves of devices based on different ILs. (a) Different anions, (b) cations with different alkyl chain length. The sensor channel size (length 32 mm, width 1.5 mm, height 2 mm).

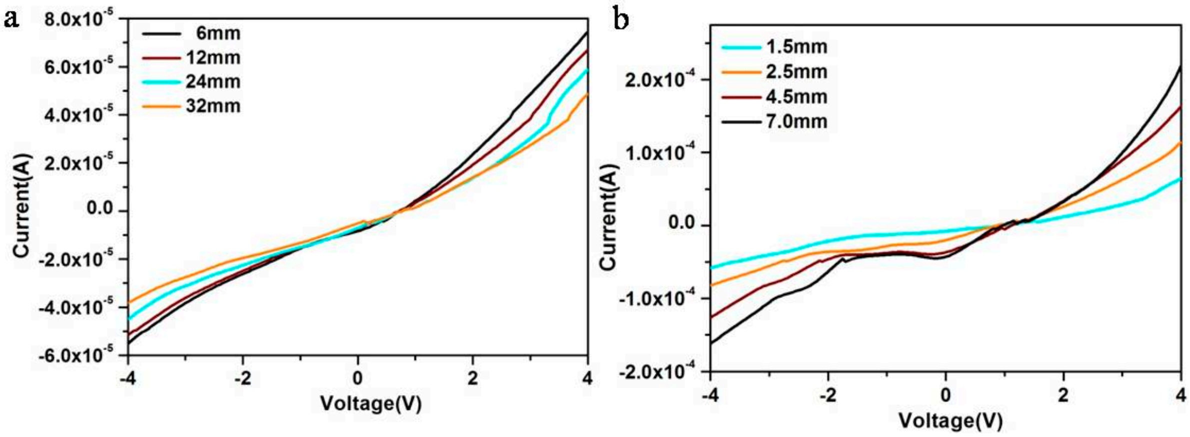

Figure S5. (a) The I-V curves of the sensors with different channel lengths (The channel width 1.5 mm, height 2 mm). (b) The I-V curves of the sensors with different channel widths (The channel length 32 mm, height 2 mm).

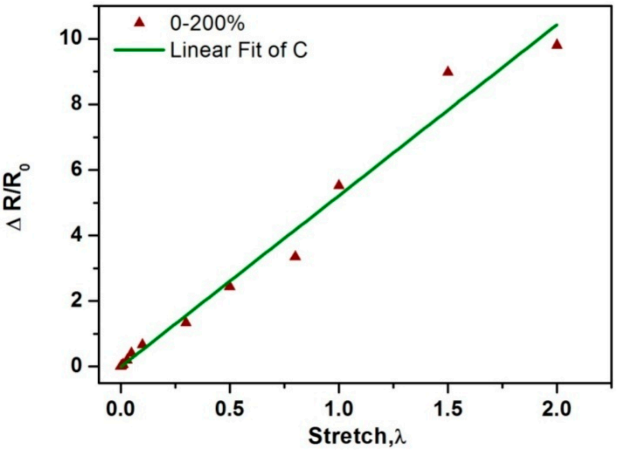

Figure S6. The relatiionship of the relative resistance change to the stretch (λ=L/L_0)

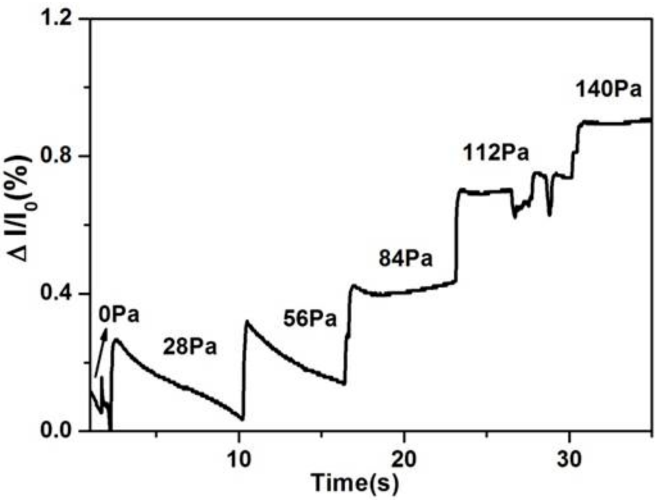

Figure S7. The relative current changes of the sensor under different pressures. The sensor channel size (length 32 mm, width 1.5 mm, height 2 mm).

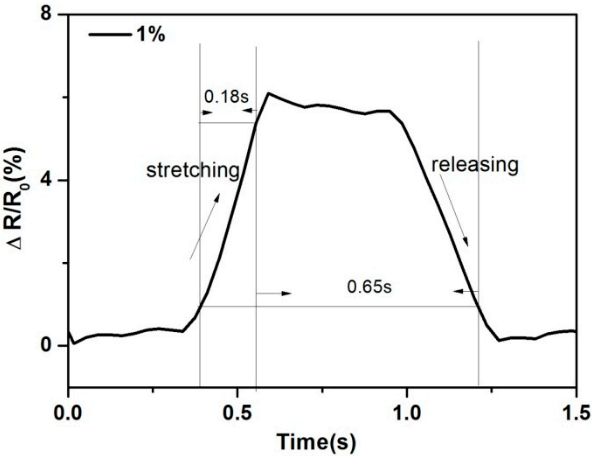

Figure S8. The relative resistance change in one stretching cycle under stain 1% at input voltage of 4V.

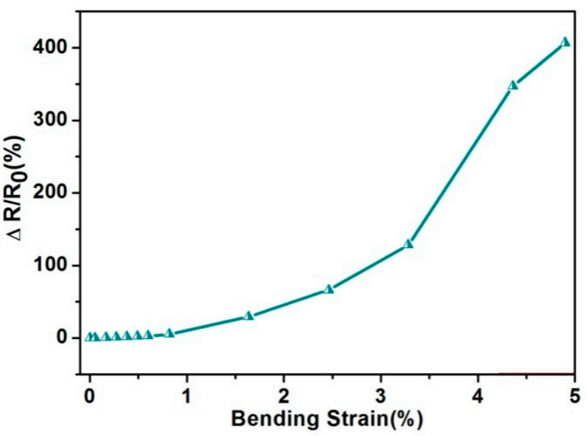

Figure S9. The relative resistance change under different bending strain at input voltage of 4V.

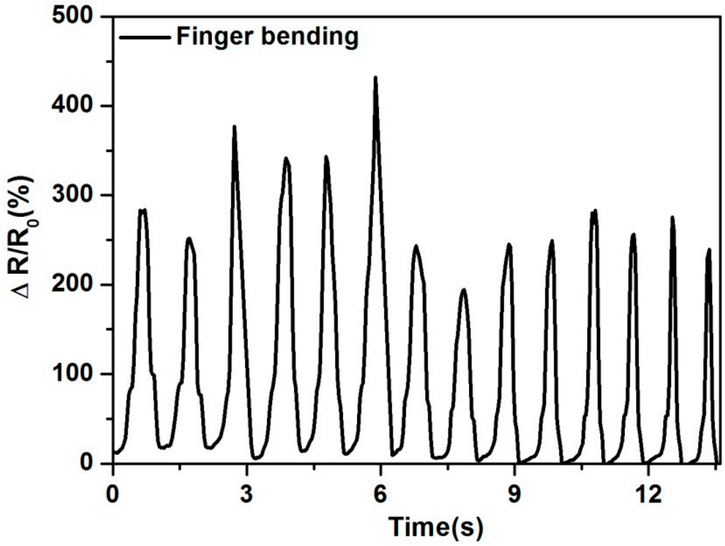

Figure S10. The relative resistance change for monitoring of the finger joint movement at input voltage of 4V. Inset: the photograph of sensor on the finger joint.

Table 1. The comparing results of reported literature and this work.

Sensing mechanism	Strain range(%)	Gause factor(Gf)	Response Time	Stability	Working voltage	Reference
piezo-resistive	0.1-500%	3	-	5000 cycles (5%、100%)	3V	[23]
piezo-resistive	25-350%	40	<3s	-	-	[25]
piezo-resistive	10-200%	40	-	2 weeks	1V	[26]
piezo-resistive	10%	2-2.5	-	-	-	[28]
piezo-resistive	0.01-350%	9.9	22ms	2 months	5V	[31]
piezo-resistive	5-60%	1.3	2.5s	100 cycles (60%)	-	[34]
capacitive	1.5-100%	0.99	12s	3000 cycles (100%)	-	[35]
piezo-resistive	1.5-60%	12.26	2.5s	5000 cycles (30%)	V_{ds}=0.7V V_{gs}=0.2V	[36]
piezo-resistive	5-80%	18.42	<100ms	1000 cycles (40%)	-	[37]
piezo-resistive	0.1-400%	7.9	92ms	1500 cycles (100%)	4V	This work(*)
